# Effects of autologous platelet-rich plasma on endometrial expansion in patients undergoing frozen-thawed embryo transfer: A double-blind RCT

**DOI:** 10.18502/ijrm.v17i6.4816

**Published:** 2019-07-29

**Authors:** Leila Nazari, Saghar Salehpour, Sedighe Hoseini, Shahrzad Zadehmodarres, Eznoallah Azargashb

**Affiliations:** ^1^Department of Obstetrics and Gynecology, School of Medicine, Preventative Gynecology Research Center, Shahid Beheshti University of Medical Sciences, Tehran, Iran.; ^2^Department of Health and Social Medicine, Shahid Beheshti University of Medical Sciences, Tehran, Iran.

**Keywords:** Platelet-rich plasma, Endometrium, Embryo transfer, Randomized controlled trial.

## Abstract

**Background:**

Adequate endometrial growth is principal for implantation and pregnancy. Thin endometrium is associated with lower pregnancy rate in assisted reproductive technology. Some frozen-thawed embryo transfer cycles are cancelled due to inadequate endometrial growth.

**Objective:**

To assess the effectiveness of autologous platelet-rich plasma (PRP) intrauterine infusion for the treatment of thin endometrium.

**Materials and Methods:**

A total of 72 patients who had a history of cancelled frozen-thawed embryo transfer cycle due to the thin endometrium (< 7mm) were assessed for the eligibility to enter the study between 2016 and 2017. Twelve patients were excluded for different reasons, and 60 included patients were randomly assigned to PRP or sham-catheter groups in a double-blind manner. Hormone replacement therapy was administered for endometrial preparation in all participants. PRP intrauterine infusion or shamcatheter was performed on day 11-12 due to the thin endometrium and it was repeated after 48 hr if necessary.

**Results:**

Endometrial thickness increased at 48 hr after the first intervention in both groups. All participants needed second intervention due to an inadequate endometrial expansion. After second intervention, endometrial thickness was 7.21 ± 0.18 and 5.76 ± 0.97 mm in the PRP group and sham-catheter group, respectively. There was a significant difference between the two groups. (p < 0.001). Embryo transfer was done for all patients in PRP group and just in six cases in the sham-catheter group. Chemical pregnancy was reported in twelve cases in the PRP group and two cases in the sham-catheter group.

**Conclusion:**

According to this trial, PRP was effective in endometrial expansion in patients with refractory thin endometrium.

## 1. Introduction

Implantation and pregnancy are influenced by two main factors, embryo and endometrium. A healthy embryo and receptive endometrium are necessary for implantation. Several treatments are used for endometrial preparation in frozen-thawed embryo transfer (FET) cycles (1). Endometrial pattern and thickness are evaluated by ultrasound examination. There is no agreement about the most recipient endometrium, however, most clinicians prefer endometrial thickness more than 7 mm for embryo transfer (2-5). Some FET cycles are canceled due to inadequate endometrial growth despite several treatments. Different protocols such as extended estrogen treatment, Pentoxifylline, low-dose Aspirin, Tamoxifen, vaginal Sildenafil, intrauterine perfusion with granulocyte-colony stimulating factor, and platelet-rich plasma (PRP) have been performed for the management of thin endometrium, but there is little consensus on the most effective one (6-14). PRP intrauterine infusion is a new approach for the treatment of refractory thin endometrium, which can stimulate proliferation and angiogenesis with a large number of growth factors and cytokines (7-11).PRP is achieved easily from an autologous blood sample that eliminates the risk of immunological reactions and transmission infections with low cost.

The aim of this double-blind randomized sham-controlled trial was to evaluate the effectiveness of PRP intrauterine infusion for the treatment of thin endometrium.

## 2. Materials and Methods

### Study population

**Figure 1:**
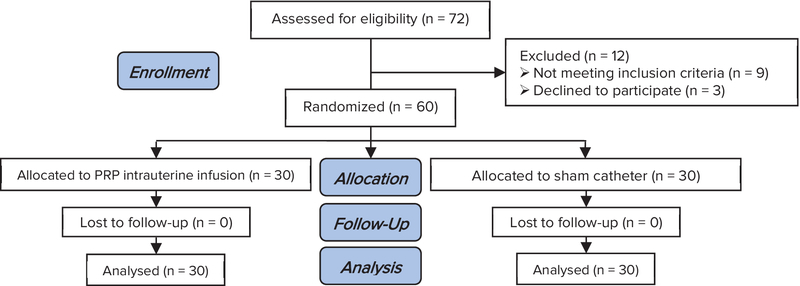
Consort flow diagram.

A total of 72 woman who had a history of cancelled FET cycle due to inadequate endometrial growth (≤ 7 mm) despite standard treatments were assessed for the eligibility to enter the study between 2016 and 2017. The inclusion criteria were age ≤ 38 yr and body mass index ≤ 30 kg/m${}^{2}$. The exclusion criteria were uterine abnormalities, hormonal disorders, and hematological disorders. All participants were subjected to the laboratory evaluation of hormonal and hematological disorders. The hysteroscopic examination was performed before the cycle if it was not previously done. Twelve women were excluded for different reasons; sixty were included in the study (Figure 1).

### Study design

This was a single-center, double-blind randomized sham-controlled trial (with balanced randomization), which was conducted in the IVF Center, Taleghani Educational Hospital, Tehran, Iran. Women were randomly selected to one of the two different groups according to the method of treatment: PRP or sham_catheter. Patients, outcome assessor, and statistician were blinded as to randomization. Randomization was carried out using computer-generated simple random tables in a 1:1 ratio. According to our pilot study the sample size was calculated after the consideration of type 1 statistical error < 5%; and type 2 statistical error < 20% based on the expected difference of endometrial thickness between the two groups. Hormone replacement therapy was administered for endometrial preparation in all patients: estradiol valerate (Aburaihan Co., Tehran, Iran) 6 mg/d was started on the 2nd or 3rd day of the menstrual cycle and that was increased to 8 mg/d on day 9-10 due to inadequate endometrial growth (≤ 7 mm). Intrauterine infusion of PRP or sham_catheter was performed on day 11-12 in intervention or control group due to the thin endometrium (thickness less than 7 mm), and it was repeated after 48 hr if required. PRP infusion or sham_catheter was performed under an ultrasound guidance. During this cycle, whenever endometrial thickness increased more than 7 mm, suppository progesterone (Cyclogest; Actavis, the UK limited, England) 400 mg twice a day was started, and ET was carried out on cleavage stage (on embryonic day 3). Estradiol valerate and progesterone supplementation were continued for next 2 weeks after ET and until 12 weeks of gestation in pregnancy. Transvaginal ultrasound was carried out by an expert gynecologist with a fellowship in infertility by one machine. Endometrial thickness was measured at the thickest part in the longitudinal axis of the uterus. PRP was prepared from autologous blood using a two-step centrifuge process; as the same protocol as reported in our pilot study (8). Then, 0.5 ml of PRP was infused into the uterine cavity with the IUI catheter (Takwin, Iran) under ultrasound guidance. The dosage and timing of PRP infusion were determined according to the pilot studies that were published in reproductive medicine.

### Outcome assessment

Transvaginal ultrasound examination was done 48 hr after PRP infusion or sham_catheter to evaluate the endometrial thickness.

### Ethical consideration

All patients signed an informed written consent. The study was approved by the ethical committee of Shahid Beheshti University of medical sciences, Tehran, Iran (IR.SBMU.MSP.REC.1394.92).

### Statistical analysis

Results were given as mean ± SD. Statistical analysis was performed using the SPSS 21.0 software package (Statistical Package for the social sciences, SPSS Inc, Chicago, IL, USA). According to the distribution of data, *t*-test and chi-square test were performed. A p < 0.05 was considered significant. The primary outcome was endometrial expansion and secondary outcomes were the chemical and clinical pregnancy that was determined by a positive serum βHCG and the presence of fetal heart beat in transvaginal ultrasound, 2 wk and 5 wk after ET, respectively.

## 3. Results

### Baseline characteristics

Sixty consenting participants were enrolled in this study who fulfilled the entry criteria and abled to complete the study and their data were analyzed. Table I shows a summary of the baseline characteristics of both groups. In term of age, BMI, and etiology of infertility there were no significant differences between the groups.

### Outcomes

Table II summarized the endometrial thickness before and after PRP or sham-catheter intervention. All participants needed PRP or sham-catheter intervention in the treatment cycles due to inadequate endometrium growth on day 11-12. As the time of first intervention, the endometrial thickness was 4.92 ± 0.67 and 5.06 ± 0.82 mm in the PRP group and sham-catheter group, respectively. There was no significant difference between the two groups (p = 0.613). After the first intervention, endometrial thickness was increased in both group; 5.99 ± 0.7 and 5.45 ± 0.82 mm in the PRP group and sham-catheter group, respectively. There was no significant difference between the two groups (p = 0.63). All participants needed second intervention 48 hr after the first one due to the inadequate endometrial thickness. After second intervention, endometrial thickness was 7.21 ± 0.18 and 5.76 ± 0.97 mm in the PRP group and sham-catheter group, respectively. There was a significant difference between the two groups (p < 0.001). FET cycle was cancelled in 24 patients in the sham-catheter group due to the persistent thin endometrium (endometrial thickness less than 7 mm) 48 hr after second intervention. Embryo transfer was done for all patients in the PRP group and just in six cases in the sham-catheter group. Chemical pregnancy was reported in twelve cases in the PRP group and two cases in the sham-catheter group (p = 0.031). Clinical pregnancy was reported in ten cases in the PRP group and one case in the sham-catheter group (p = 0.048).

**Table 1:** Baseline characteristics


	**PRP (n = 30)**	**Sham (n = 30)**	**p-value**
Age (yr)*	33.93 ± 2.76	32.33 ± 4.79	0.274
BMI (kg)*	24.3 ± 2.24	25.46 ± 2.68	0.262
Etiology of Infertility**			
Male Factor	6 (20)	8 (26.6)	
DOR	4 (13.3)	2 (6.6)	
Tubal Factor	2 (6.6)	2 (6.6)	
Anovulation	6 (20)	4 (13.3)	
Mixed	12 (40)	14 (46.6)	0.499
Note: *Data presented as mean ± SD; **data presented as n (%)			
No staticalsignificancewas observed between the two groups:			
BMI: Body mass index DOR: Diminished ovarian reserve			

**Table 2:** Outcomes including endometrial thickness and pregnancy rate


	**PRP (n = 30)**	**Sham (n = 30)**	**p-value**
Endometrial thickness (mm)*			
before intervention	4.92 ± 0.671	5.06 ± 0.821	0.613
after 1${}^{st}$ intervention	5.993 ± 0.701	5.453 ± 0.823	0.63
after 2${}^{nd}$ intervention	7.213 ± 0.188	5.767 ± 0.973	< 0.001$
Chemical pregnancy**	12 (40)	2 (6.7)	0.031$
Clinical pregnancy**	10 (33.3)	1 (3.3)	0.048$
Note: *data presented as mean ± SD; **Data presented as n (%); $: Statically significant; SD: Standard deviation; PRP: Platelet-rich plasma			

## 4. Discussion

Adequate endometrial growth is principal for embryo implantation and pregnancy. According to the literature, the incidence of the thin endometrium is about 2.5% in in-vitro fertilization (IVF) cycles which can lead to cycle cancellation. Also, an endometrial thickness < 7 mm is associated with lower pregnancy rate in IVF cycles. Besides the important role of endometrial thickness for implantation, there is some evidence about its effects on neonatal birth weight in assisted reproductive technology (15-17). Intrauterine infusion of PRP to increase of endometrial thickness was first proposed by Change and colleagues in 2015 (7). Successful endometrial growth was reported in all of five patients with a history of refractory thin endometrium. Recent reports from our center have recommended that intrauterine infusion of PRP may be effective in the improvement of endometrial growth, expansion and implantation. In our pilot trial, 10 patients who had a history of refractory thin endometrium were recruited in the study and PRP intrauterine infusion was administered. Endometrial thickness increased during 48 hours after first PRP and reached > 7 mm after second PRP. Embryo transfer was carried out for all patients. Five chemical pregnancies and four live births were reported (7, 8, 18). Another pilot study revealed the role of PRP in the improvement of endometrial thickness and vascularity in women with suboptimal endometrial thickness and reduced the incidence of cycle cancellation (9). Two recent studies suggested PRP intrauterine infusion for two novel approaches in assisted reproductive technology. These papers revealed the effective role of PRP in regeneration in Asherman's syndrome and the positive effects of PRP in the improvement of pregnancy outcome in repeated implantation failure (11, 18). PRP is an autologous fraction of the blood that contains a large amount of platelet, growth factors and cytokines such as vascular endothelial growth factor, platelet-derived growth factor, epidermal growth factor, fibroblast growth factor, insulin-like growth factor I, II, transforming growth factor, interleukin 8, hepatocyte growth factor, and connective tissue growth factor. Recently, the stimulating, proliferating, and tissue regenerative effects of PRP have been used in several medical conditions in orthopedics, ophthalmology, dental surgery, and wound healing. According to our pilot trials, there isn't any report of maternal, fetal, and neonatal adverse effects (8, 18-23). The result of this trial revealed the efficacy of PRP intrauterine infusion on endometrial expansion and proliferation in women with idiopathic thin endometrium. As we know, this is the first double-blind randomized sham-controlled trial that evaluated the efficacy of PRP in endometrial growth. We performed two-dimensional transvaginal ultrasound examination for measure endometrial thickness. According to the role of endometrial vascularity on implantation and pregnancy and the angiogenetic effects of PRP, it is suggested to design further studies and apply color Doppler and three-dimensional ultrasound examination for the detection of endometrial and sub-endometrial vasculature pattern and cavity volume before and after PRP infusion (24).

## 5. Conclusion

According to this trial, PRP was effective in endometrial expansion in patients with refractory thin endometrium.

##  Conflict of Interests 

No conflict of interests has been declared.
